# FGF21 and GDF15 Act Synergistically to Regulate Systemic Metabolic Homeostasis in Mice Lacking OPA1 in Thermogenic Adipocytes

**DOI:** 10.1002/oby.70004

**Published:** 2025-09-02

**Authors:** Joshua Peterson, Jayashree Jena, Ayushi Sood, Shelly Roitershtein, David Smith, Renata O. Pereira

**Affiliations:** ^1^ Fraternal Order of Eagles Diabetes Research Center and Department of Internal Medicine, Division of Endocrinology and Metabolism, Carver College of Medicine University of Iowa Iowa City Iowa USA

**Keywords:** Brown Adipose Tissue, FGF21, GDF15, Mitochondrial Stress, Obesity

## Abstract

**Objective:**

Our previous studies showed that mice lacking the mitochondrial fusion protein optic atrophy 1 (OPA1 BKO) in brown adipose tissue (BAT) have high metabolic rates and are resistant to diet‐induced obesity (DIO) via effects partially mediated by independent actions of fibroblast growth factor 21 (FGF21) and growth differentiation factor 15 (GDF15) secretion from BAT. We examined whether FGF21 and GDF15 act synergistically, contributing to the systemic metabolic adaptations reported in OPA1 BKO mice.

**Methods:**

We generated mice simultaneously lacking the *Opa1*, *Fgf21*, and *Gdf15* genes in thermogenic adipocytes (TKO) and assessed energy homeostasis and glucose metabolism after regular chow or high‐fat diet feeding.

**Results:**

Young TKO mice fed regular chow had impaired glucose tolerance, while insulin sensitivity was unchanged. Notably, combined *Fgf21* and *Gdf15* deletion in OPA1 BKO significantly blunted the resistance to DIO and insulin resistance observed in OPA1 BKO mice.

**Conclusions:**

FGF21 and GDF15 act synergistically to maintain glucose homeostasis and promote resistance to DIO in mice lacking OPA1 in BAT, highlighting the potential of combined therapies using FGF21 and GDF15 for the treatment of metabolic disorders.

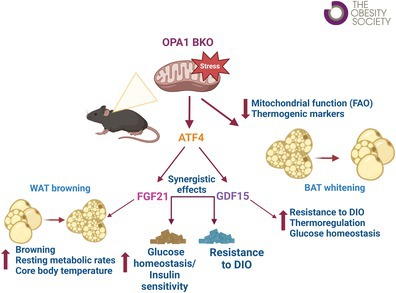

## Graphical abstract

OPA1 deletion in BAT leads to ATF4 activation, which is required to promote FGF21 and GDF15 secretion as batokines. In addition to their independent effects regulating metabolism, in the present study, we uncovered that FGF21 and GDF15 act synergistically to improve glucose homeostasis and promote resistance to diet‐induced obesity. Created in BioRender. Alambert, R. (2025) https://BioRender.com/kii6rgt.

**Study Importance**

- What is already known?
  ○ Mitochondrial stress in brown adipose tissue induces the secretion of fibroblast growth factor 21 (FGF21) and growth differentiation factor 15 (GDF15) as batokines.
  ○ FGF21 and GDF15 exert independent effects, improving glucose homeostasis and energy metabolism in mice in response to mitochondrial abnormalities and in preclinical studies.
- What does this study add?
  ○ Here we show that FGF21 and GDF15 act synergistically as batokines to maintain glucose homeostasis and insulin sensitivity in mice fed chow diet and to promote resistance to diet‐induced obesity in a mouse model of mitochondrial stress.
- How might these results change the direction of research or the focus of clinical practice?
  ○ FGF21 and GDF15 have synergistic effects mediating a mitohormetic response that improves systemic metabolic health.
  ○ Although FGF21 and GDF15 analogues in isolation have modest effects on body weight reduction in clinical studies, therapies combining FGF21 and GDF15 might be efficacious in attenuating obesity and improving cardiometabolic health.

## 1 Introduction

In recent years, the incidence of obesity and its associated diseases, such as type 2 diabetes and cardiovascular disease, have increased dramatically [1]. The discovery of active brown adipose tissue (BAT) in adult humans has attracted interest in investigating the potential of BAT activation to mitigate obesity and its complications [2]. Several studies in rodents and humans provide compelling evidence that BAT plays an important role in whole‐body energy homeostasis and glucose and lipid metabolism via its thermogenic activity [3, 4, 5, 6, 7], as well as via the release of various signaling molecules, including endocrine factors known as batokines [8].

Our recent studies in mice lacking the mitochondrial fusion protein optic atrophy 1 (OPA1) in BAT reinforce the concept that BAT secretory function is crucial to maintaining systemic metabolic homeostasis in mice [9, 10]. OPA1 BKO mice developed metabolic adaptations that increased their resting metabolic rates and improved insulin sensitivity under baseline conditions and promoted resistance to diet‐induced obesity (DIO). Our data revealed that secretion of fibroblast growth factor 21 (FGF21) and growth differentiation factor 15 (GDF15) as batokines, which was induced by the transcription factor activating transcription factor 4 (ATF4), mediated these metabolic adaptations. FGF21 action was required to promote leanness in mice fed chow diet, but was dispensable to promote resistance to DIO [9]. Conversely, GDF15 partially mediated the resistance to DIO in mice lacking OPA1 in BAT, while improving glucose homeostasis [10]. Interestingly, mice with concomitant deletion of *Atf4* and *Opa1* in BAT were unable to induce FGF21 and GDF15 and lacked the metabolic adaptations observed in OPA1 BKO mice, including resistance to DIO. These data suggest that FGF21 and GDF15 might act together downstream of ATF4 to regulate energy homeostasis and glucose metabolism in OPA1‐deficient mice [9].

In the present study, we sought to test the hypothesis that, in addition to their independent effects, FGF21 and GDF15 also act synergistically to improve systemic metabolism in OPA1‐deficient mice. We generated mice simultaneously lacking OPA1, FGF21, and GDF15 in thermogenic adipocytes and assessed baseline and diet‐induced changes in body composition, energy homeostasis, and glucose metabolism. Our results reveal that FGF21 and GDF15 have synergistic effects that maintain glucose homeostasis and promote resistance to DIO in mice lacking OPA1 in BAT. These studies highlight the potential of combined therapies using FGF21 and GDF15 for the treatment of metabolic disorders.

## 2 Methods

### 2.1 Mouse Models

Experiments were performed in male and/or female mice on a C57Bl/6J background. *Opa1* ^fl/fl^ [11], *Fgf21* ^fl/fl^ [12], *Gdf15* ^fl/fl^ [10], and *Atf4* ^fl/fl^ mice [13] were generated as previously described. Transgenic mice expressing Cre recombinase under the control of the *Ucp1* promoter (Tg [Ucp1‐cre]1Evdr) [14] were acquired from the Jackson Laboratory (Bar Harbor, ME, #024670). Compound mutants were generated by crossing OPA1^fl/fl^ mice harboring *Ucp1* Cre with FGF21^fl/fl^ and GDF15^fl/fl^ for generation of OPA1/FGF21/GDF15 triple knockout mice (TKO) or with ATF4^fl/fl^ mice for generation of OPA1/ATF4 double knockout mice (DKO), as previously described [9]. Wild type (WT) controls for each compound mutant were mice harboring the respective homozygous floxed alleles but lacking *Ucp1* Cre. Mice were weaned at 3 weeks of age and kept on standard chow (2920X Harlan Teklad, Indianapolis, IN). For DIO studies, 6‐week‐old mice were fed a high‐fat diet (HFD; 60% of kilocalories from fat; Research Diets, New Brunswick, NJ, D12492) for 12 weeks. After 11 weeks of HFD feeding, male mice were placed in a Promethion System (Sable Systems International, Las Vegas, NV) for metabolic activity measurements. Unless otherwise noted, animals were housed at 22°C with a 12‐h light, 12‐h dark cycle with free access to water and standard chow or HFD. A subset of OPA1 BKO female mice were reared at thermoneutrality (30°C), until they were ~8 weeks of age. All mouse experiments presented in this study were conducted in accordance with NIH animal research guidelines and were approved by the University of Iowa IACUC.

### 2.2 GTT, ITT, Nuclear Magnetic Resonance, and Serum Analysis

Glucose tolerance tests (GTT) were performed after a 4‐h fast for baseline studies and a 6‐h fast for DIO studies, after 10 weeks of HFD feeding. Mice were administered glucose intraperitoneally (1 g/kg body weight), as described [15]. Insulin tolerance tests (ITT) were performed following a 2‐h fast by injecting insulin intraperitoneally (0.75 U/kg body weight; Humulin, Eli Lilly, Indianapolis, IN) after 12 weeks of HFD feeding. Blood glucose was determined using a glucometer at regular time intervals (Glucometer Elite, Bayer, Tarrytown, NY). Tissue‐specific insulin signaling was assessed following a 16‐h fast with tissues collected for immunoblotting 20 min after insulin (2 U/kg body weight) or 0.9% saline was injected intraperitoneally [16]. Insulin and glucose solutions were prepared in sterile 0.9% saline and dosed based on body weight. Plasma insulin was measured after a 6‐h fast using a commercially available kit according to the manufacturer's directions (Ultra‐Sensitive Mouse Insulin ELISA Kit, Chrystal Chem, Downers Grove, IL). Serum FGF21 (BioVendor ELISA kit, Asheville, NC) and GDF15 (R&D Systems, Minneapolis, MN) were measured under ad libitum‐fed conditions using commercially available kits according to the manufacturer's directions. Whole‐body composition was measured by nuclear magnetic resonance in a Bruker Minispec NF‐50 instrument https://doi.org (Billerica, MA).

### 2.3 Analysis of Triglyceride Levels

Hepatic triglyceride levels were measured in young mice fed control chow (baseline studies) or HFD for 12 weeks using the EnzyChrom Triglyceride Assay Kit (BioAssay Systems, Hayward, CA), as previously described [9, 17].

### 2.4 RNA Extraction and Quantitative RT‐PCR

Total RNA was extracted from tissues with TRIzol reagent (Invitrogen, Waltham, MA) and purified with the RNeasy kit (QIAGEN Inc., Germantown, MD). Quantitative RT‐PCR was performed as previously described [9]. Data were normalized to *Tbp* expression, and results are shown as relative mRNA levels. Quantitative PCR primers were designed using Primer‐Blast or previously published sequences [18]. Primer sequences are listed in Table 1.

**TABLE 1:** Primer sequences.

Gene name	Forward	Reverse
*Fgf21*	TGACGACCAAGACACTGAAGC	TTTGAGCTCCAGGAGACTTTCTG
*Ucp1*	GTGAAGGTCAGAATGCAAGC	AGGGCCCCCTTCATGAGGTC
*Tbp*	TCTGGAATTGTACCGCAGCTT	CTGCAGCAAATCGCTTGGGA
*Dio2*	AATTATGCCTCGGAGAAGACCG	GGCAGTTGCCTAGTGAAAGGT
*Ppargc1α*	GTAAATCTGCGGGATGATGG	AGCAGGGTCAAAATCGTCTG
*Gdf15*	GAGAGGACTCGAACTCAGAAC	GACCCCAATCTCACCTCTG
*Gapdh*	AACGACCCCTTCATTGAC	TCCACGACATACTCAGCAC

### 2.5 Western Blot Analysis

Immunoblotting analysis was performed as previously described [19]. Approximately 30 mg of frozen BAT, inguinal white adipose tissue (iWAT), liver, or skeletal muscle was homogenized in 150 μL of lysis buffer, as previously described [9]. HALT protease/phosphatase inhibitors (Thermo Fisher Scientific, Waltham, MA) were added to the lysis buffer immediately before use, and samples were processed using the TissueLyser II (QIAGEN). Tissue lysates were resolved on SDS‐PAGE and transferred to nitrocellulose membranes (Millipore Corp., Billerica, MA). Membranes were incubated with primary antibodies overnight and with secondary antibodies for 1 h at room temperature.

### 2.6 Antibodies

The following primary antibodies were used: UCP1 (1:1000, Abcam, Waltham, MA, Ab10983), tyrosine hydroxylase (TH) (1:1000, Cell Signaling Technology, Sanvers, MA, #2792), pAKT (1:1000, Cell Signaling Technology, # 4060S), AKT (pan) (11,000, Cell Signaling Technology, #2920), IRDye 800CW anti‐Mouse (1:10,000, LI‐COR, Lincoln, NE, #925‐32212), and Alexa Fluor anti‐rabbit 680 (1:10,000, Invitrogen, #A27042). Fluorescence was quantified using an Odyssey imager (LICORbio, Lincoln, NE).

### 2.7 Mitochondrial Isolation

Mitochondrial fraction was isolated from BAT as previously described [9, 20]. Briefly, tissue was excised, rinsed in ice‐cold PBS, and maintained in ice‐cold isolation buffer (500 mM EDTA, 215 mM D‐mannitol, 75 mM sucrose, 0.1% free fatty acid bovine serum albumin [BSA], 20 mM HEPES, pH 7.4 with KOH) until ready for homogenization. Bradford assay was performed to determine protein concentration.

### 2.8 Oxygen Consumption

Mitochondrial oxygen consumption rates were assessed in 50 μg of mitochondrial protein using the Oroboros O2K Oxygraph system (Oroboros Instruments, Innsbruck, Austria) in buffer Z containing (mM) 110 K‐MES, 35 KCl, 1 EGTA, 5 K_2_HPO_2_, 3 MgCl_2_·6H_2_O, and 5‐mg/mL BSA (pH 7.4, 295 mOsm/L). The following substrates and nucleotides were utilized: pyruvate (5 mM) + malate (2 mM), followed by the addition of ADP (5 mM).

### 2.9 Histology

Fragments of BAT and iWAT from ~12‐week‐old mice were embedded in paraffin, portioned into 4‐μm‐thick sections, and stained with hematoxylin–eosin (H&E) (Fisher, Pittsburgh, PA). Light microscopy was performed using an Olympus BX63 (Olympus, Shinjuku, Tokyo, Japan) [10].

### 2.10 Data Analysis

Unless otherwise noted, all data are reported as mean ± SEM. To determine statistical differences, Student's *t*‐test was performed for comparison of two groups; one‐way ANOVA followed by Tukey's multiple‐comparison test was utilized with three or more groups; and two‐way ANOVA followed by Tukey's multiple‐comparison test was utilized to compare three or more groups when two independent variables were taken into consideration. A probability value of *p* ≤ 0.05 was considered significantly different. Statistical calculations were performed using GraphPad Prism (La Jolla, CA). CalR software was used to extract averaged data for metabolic parameters measured in the Promethion system for both the light and dark cycles [21].

## 3 Results

### 3.1 Deletion of *Fgf21* and *Gdf15* in OPA1 BKO Mice Prevents the Increase of GDF15 and FGF21 Serum Levels

**FIGURE 1:**
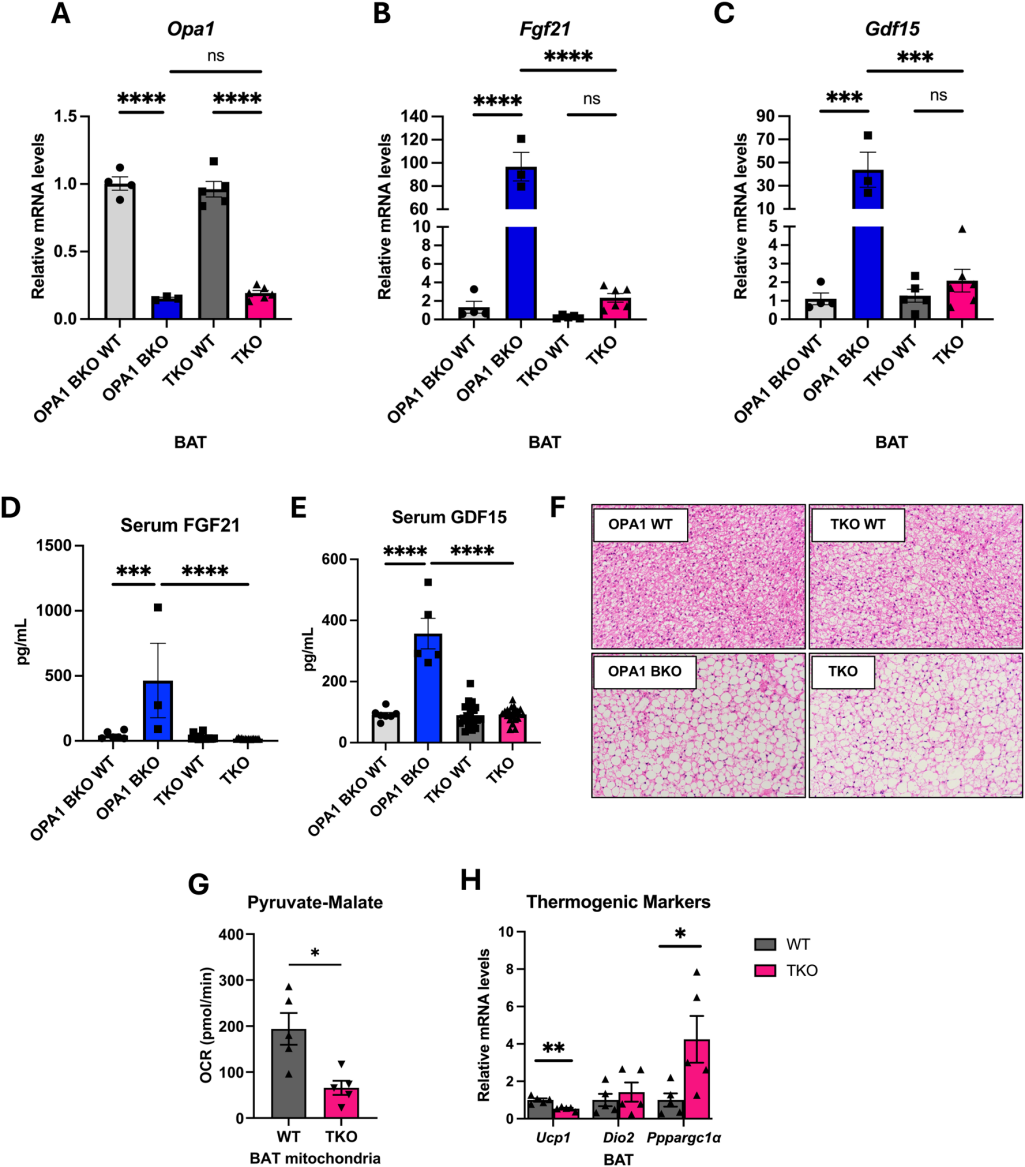
TKO mice lack increase in GDF15 and FGF21 serum levels while maintaining impairment in mitochondrial respirations. Data collected in 8‐week‐old male OPA1 BKO and TKO and respective WT control mice fed chow diet ([panels A–C]: *n* = 4 OPA1 BKO WT; *n* = 3 OPA1 BKO; *n* = 5 TKO WT; *n* = 6 TKO). (A) Relative mRNA expression of *Opa1* in BAT normalized to tata box protein (*Tbp*) expression. (B) Relative mRNA expression of *Fgf21* in BAT normalized to *Tbp* expression. (C) Relative mRNA expression of *Gdf15* in BAT normalized to *Tbp* expression. (D) Serum levels of FGF21 in ad libitum‐fed mice (*n* = 6 OPA1 BKO WT; *n* = 3 OPA1 BKO; *n* = 15 TKO WT; *n* = 14 TKO). (E) GDF15 serum levels in ad libitum‐fed mice (*n* = 7 OPA1 BKO WT; *n* = 5 OPA1 BKO; *n* = 21 TKO WT; *n* = 20 TKO). (F) Representative BAT sections stained with H&E. Scale bar = 50 μm. (G) ADP‐stimulated (state 3) pyruvate/malate‐supported oxygen consumption rates (OCR) in mitochondria isolated from BAT (*n* = 5 WT/TKO). (H) mRNA expression of thermogenic genes normalized to *Tbp* expression (*n* = 5 WT/TKO). Data expressed as mean ± SEM. Significant differences were determined by one‐way ANOVA followed by Tukey's multiple‐comparison test or Student's *t*‐test using a significance level of *p* ≤ 0.05. *p ≤ 0.05, ***p ≤ 0.001, ****p ≤ 0.0001.

To validate deletion of *Opa1*, *Fgf21*, and *Gdf15* in BAT of TKO mice, we performed quantitative RT‐PCR. *Opa1* mRNA levels were similarly reduced in OPA1 BKO and TKO mice (Figure 1A). Importantly, mRNA levels of *Fgf21* (Figure 1B) and *Gdf15* (Figure 1C) were both reduced in BAT of TKO compared to OPA1 BKO mice. OPA1 deletion in BAT resulted in significant induction of FGF21 (Figure 1D) and GDF15 (Figure 1E) serum levels, which were completely normalized in TKO mice, confirming that *Opa1* deletion in BAT induces FGF21 and GDF15 secretion as batokines [9, 10]. Histologically, both OPA1 BKO and TKO mice had comparable levels of BAT whitening relative to their respective WT controls, as shown by larger adipocytes with unilocular lipid droplets (Figure 1F). *Opa1* deletion in BAT resulted in impaired mitochondrial respiratory capacity [9]. To determine whether this phenotype is affected in TKO mice, we measured mitochondrial oxygen consumption rates in isolated mitochondria from BAT. As reported for OPA1 BKO mice [9], our data showed reduced pyruvate/malate‐supported respirations in TKO mice (Figure 1G). Similarly, expression of thermogenic markers in BAT showed repression of *Ucp1* mRNA levels, while *Ppargc1α* levels were induced in TKO mice relative to WT controls (Figure 1H), mirroring our data in OPA1 BKO mice (Figure S1A). Together, these findings suggest that the mitochondrial dysfunction and thermogenic gene profile observed in OPA1 BKO mice are maintained when FGF21 and GDF15 are deleted. Importantly, we previously showed that OPA1 BKO female mice reared at thermoneutrality maintain high resting metabolic rates and are leaner than their WT littermate controls [9]. Here we show that even at thermoneutrality, OPA1 BKO mice experience similar transcriptional changes in BAT, including reduced expression of thermogenic genes (Figure S1B) and induction of *Fgf21* and *Gdf15* transcripts (Figure S1C,D), resulting in increased circulating levels (Figure S1E,F). Moreover, compensatory browning of white adipose tissue (WAT) is also preserved under these conditions (Figure S1G). Together, these findings strongly indicate that the metabolically favorable phenotype observed in OPA1 BKO mice occurs independently of BAT thermogenic activity and ambient temperature.

### 3.2 Young TKO Mice Develop Glucose Intolerance and Lose Improvement in Insulin Sensitivity Observed in OPA1 BKO Mice Fed Regular Chow

**FIGURE 2:**
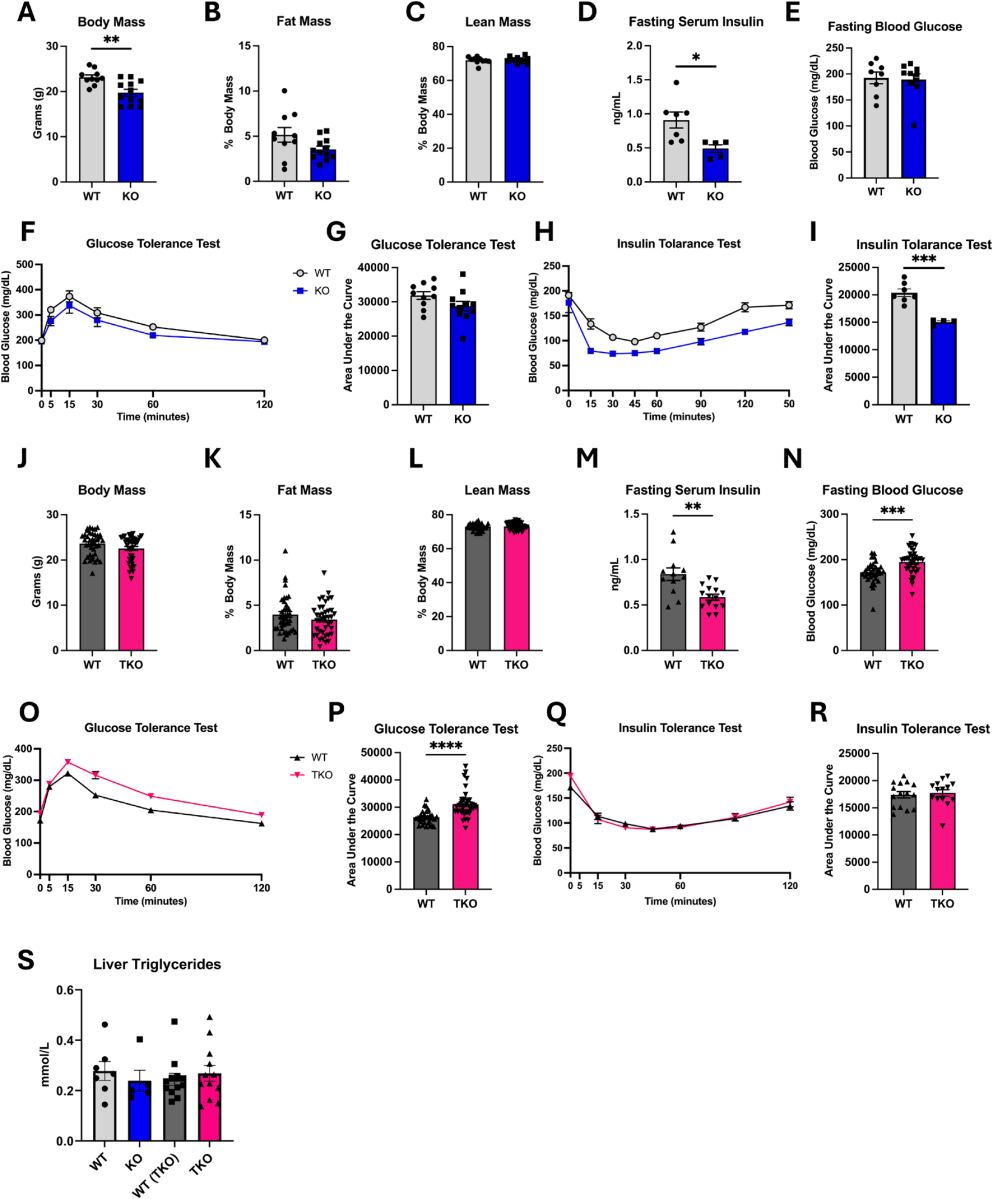
Young TKO mice develop glucose intolerance and lose improvement in insulin sensitivity observed in OPA1 BKO mice fed regular chow. In panels A–I and S, data collected in 8‐week‐old male OPA1 BKO and WT control mice fed chow diet ([panels A–C]: *n* = 10 WT; *n* = 12 OPA1 BKO). (A) Body mass. (B) Percent fat mass to body weight. (C) Percent lean mass to body weight. (D) Fasting serum insulin levels (*n* = 7 WT; *n* = 5 OPA1 BKO). (E) Fasting blood glucose levels (*n* = 10 WT; *n* = 11 OPA1 BKO). (F) Glucose tolerance test (*n* = 10 WT; *n* = 11 OPA1 BKO). (G) AUC for glucose tolerance test (*n* = 10 WT; *n* = 11 OPA1 BKO). (H) Insulin tolerance test (*n* = 7 WT; *n* = 4 OPA1 BKO). (I) AUC for insulin tolerance test (*n* = 7 WT; *n* = 4 OPA1 BKO). In panels J–S, data collected in 8‐week‐old male TKO and WT control mice fed chow diet ([panels J–L]: *n* = 40 WT; *n* = 41 TKO). (J) Body mass. (K) Percent fat mass to body weight. (L) Percent lean mass to body weight. (M) Four‐h fasting insulin levels (*n* = 12 WT; *n* = 15 TKO). (N) Four‐h fasting blood glucose (*n* = 32 WT; *n* = 35 TKO). (O) Glucose tolerance test (*n* = 32 WT; *n* = 35 TKO). (P) AUC for glucose tolerance test (*n* = 32 WT; *n* = 35 TKO). (Q) Insulin tolerance test (*n* = 15 WT; *n* = 14 TKO). (R) AUC for insulin tolerance test (*n* = 15 WT; *n* = 14 TKO). (S) Liver triglycerides (*n* = 7 OPA1 BKO WT; *n* = 5 OPA1 BKO; *n* = 12 TKO WT/TKO). Data expressed as mean ± SEM. Significant differences determined by one‐way ANOVA followed by Tukey's multiple‐comparison test or Student's *t*‐test using a significance level of *p* ≤ 0.05. *p ≤ 0.05, **p ≤ 0.01, ***p ≤ 0.001, ****p ≤ 0.0001.

We have previously shown that OPA1 BKO mice have reduced body mass when fed regular chow [9]. Here, we recapitulated these findings by conducting baseline metabolic phenotyping in young (~8 weeks of age) OPA1 BKO mice. As previously reported, 8‐week‐old OPA1 BKO male mice fed regular chow have reduced body mass (Figure 2A), with no change in percent fat mass (Figure 2B) or percent lean mass (Figure 2C) but significantly reduced total fat (Figure S2A) and lean mass (Figure S2B). Fasting serum insulin levels (Figure 2D) were reduced in OPA1 BKO mice, while no changes in fasting blood glucose levels (Figure 2E) or GTT (Figure 2F,G) were observed. Moreover, here we show for the first time that insulin sensitivity is improved under baseline conditions in OPA1 BKO mice, as indicated by ITT (Figure 2H,I).

Contrary to what we observed in OPA1 BKO mice, age‐matched male TKO mice had no differences in body mass or body composition when compared to WT littermate controls (Figure 2J–L, Figure S2F,G). TKO mice also had no changes in liver, BAT, iWAT, or gonadal white adipose tissue (gWAT) tissue weight (Figure S2H–K). Surprisingly, similar to OPA1 BKO mice, fasting insulin levels were significantly reduced in TKO mice (Figure 2M), whereas fasting blood glucose levels were elevated (Figure 2N) and glucose tolerance was impaired (Figure 2O,P), revealing impaired glucose homeostasis. The improvement in insulin sensitivity in OPA1 BKO mice was lost in the TKO animals, as shown by similar areas under the curve (AUC) for ITT (Figure 2Q,R). These changes in glucose homeostasis occurred despite no changes in hepatic triglyceride accumulation between genotypes (Figure 2S). Of note, the impaired glucose homeostasis in 8‐week‐old TKO mice is a phenomenon that we did not observe when FGF21 or GDF15 was individually deleted in the OPA1 BKO background [9, 10]. We also did not see any changes in glucose tolerance (Figure S2L,M) in 20‐week‐old mice lacking both OPA1 and FGF21 in BAT (OPA1/FGF21), whereas fasting glucose levels were significantly higher in these mice (Figure S2N). Importantly, mice lacking OPA1 and activating transcription factor 4 (ATF4) in BAT lack induction in FGF21 and GDF15 levels [9, 10] and also show impaired glucose homeostasis under baseline conditions (Figure S2O–Q). Together, these data suggest that FGF21 and GDF15 act synergistically to improve glucose homeostasis and insulin sensitivity in OPA1 BKO male mice, with their combined absence resulting in glucose intolerance and prevention of improvement in insulin sensitivity. Finally, our data in 8‐week‐old TKO female mice also show no differences in body weight or body composition when fed chow diet (Figure S2R–T), but contrary to what we observed in males, glucose homeostasis was unchanged between WT and TKO female mice (Figure S2U–W).

### 3.3 Compensatory Browning of iWAT Is Prevented in TKO Mice

**FIGURE 3:**
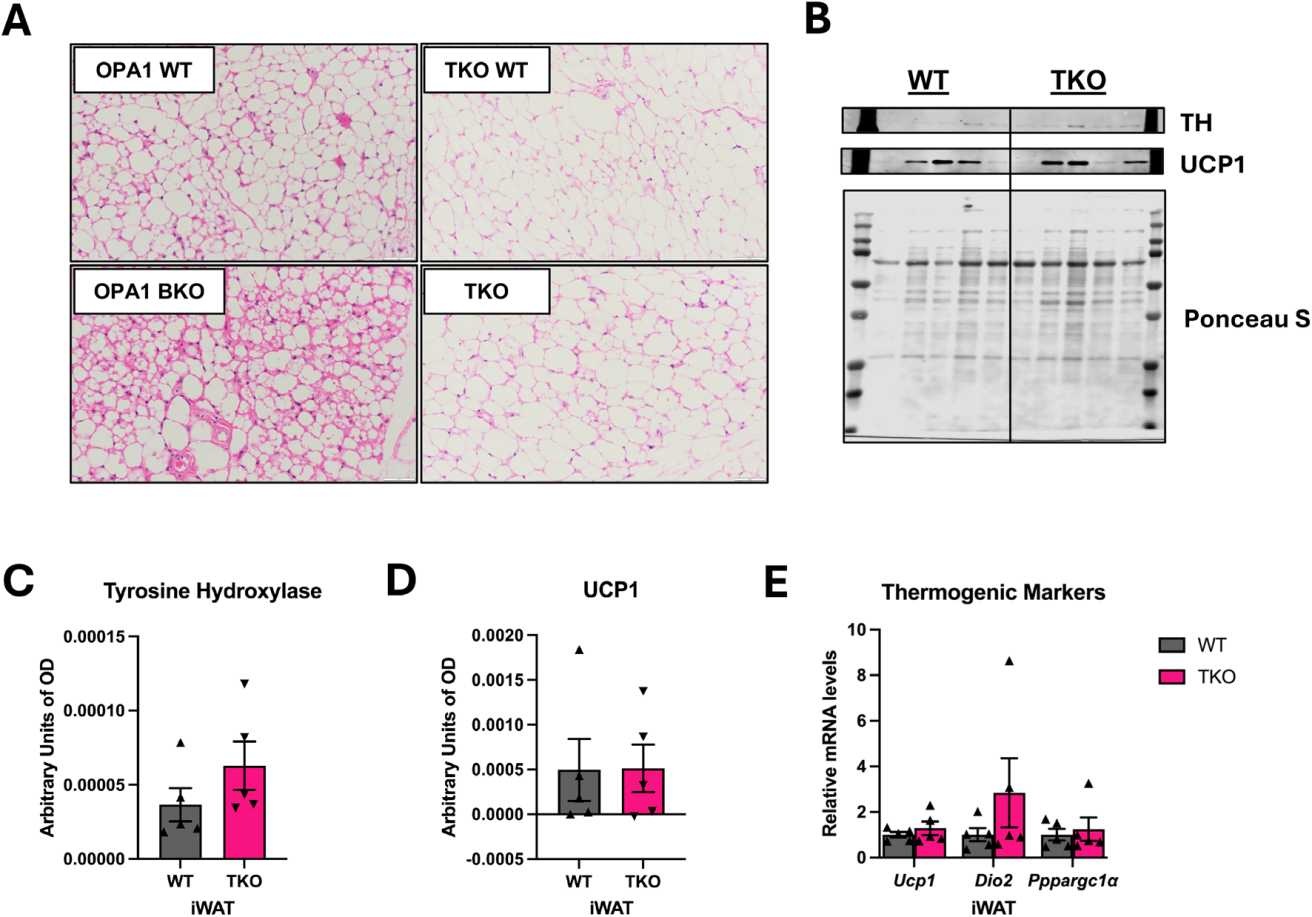
Compensatory browning of iWAT is prevented in TKO mice (*n* = 5 WT; *n* = 5 TKO). (A) Representative iWAT sections stained with H&E. Scale bar = 50 μm. (B) Immunoblot of tyrosine hydroxylase (TH) levels and uncoupling protein 1 (UCP1) in iWAT. (C) Densitometric analysis of UCP1 protein levels in iWAT normalized to Ponceau red staining. (D) Densitometric analysis of TH protein levels in iWAT normalized to Ponceau red staining. (E) mRNA expression of thermogenic genes in iWAT normalized to *Tbp* levels. Data expressed as mean ± SEM. Significant differences determined by Student's *t*‐test using a significance level of *p* ≤ 0.05.

In our previous study, we showed that OPA1 BKO mice have increased baseline browning of iWAT under room temperature conditions [9], which is maintained under thermoneutrality (Figure S1G). Our previous studies suggest that these changes in browning are primarily driven by FGF21 [9, 10]. Here we show that simultaneous deletion of FGF21 and GDF15 in BAT of OPA1 BKO mice prevented browning of iWAT, as shown by histological assessment of iWAT. While in OPA1 BKO mice we observed an increased number of brown‐like adipocytes within the iWAT, this phenotype was absent in TKO mice relative to their WT controls (Figure 3A). This was confirmed by immunoblot showing no changes in UCP1 and TH protein levels (Figure 3B–D) or in mRNA expression of thermogenic markers in iWAT of TKO mice (Figure 3E).

### 3.4 Insulin Signaling Is Unchanged in Various Tissues in Young TKO Mice Fed Regular Chow

**FIGURE 4:**
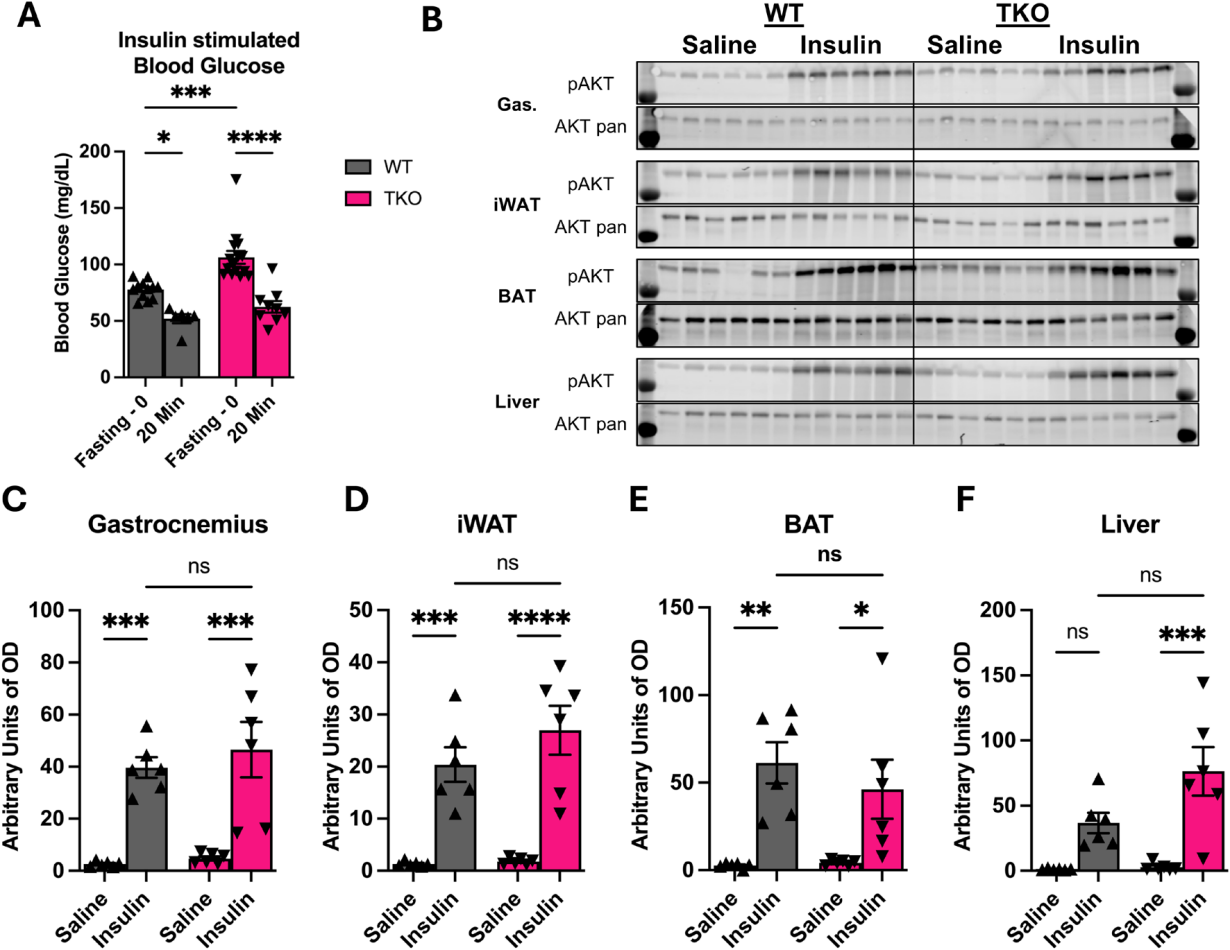
Insulin signaling is unchanged in various insulin‐responsive tissues in young TKO mice fed regular chow. Data collected in 8‐week‐old male TKO and WT control mice ([panels B–F]: *n* = 5 WT; *n* = 5 TKO). (A) Blood glucose levels after 6‐h fast (*n* = 12 WT; *n* = 15 TKO) and 20 min after insulin (*n* = 6 WT; *n* = 9 TKO) (intraperitoneal) injection. (B) Immunoblot of phosphorylated AKT (pAKT) and total AKT (AKT pan) in gastrocnemius, inguinal white adipose tissue (iWAT), brown adipose tissue (BAT), and liver. (C) Densitometric analysis of pAKT protein levels in gastrocnemius muscle normalized to total AKT protein levels. (D) Densitometric analysis of pAKT protein levels in iWAT normalized to total AKT protein levels. (E) Densitometric analysis of pAKT protein levels in BAT normalized to total AKT protein levels. (F) Densitometric analysis of pAKT protein levels in liver normalized to total AKT protein levels. Data expressed as mean ± SEM. Significant differences determined by two‐way ANOVA followed by Tukey's multiple‐comparison test using a significance level of *p* ≤ 0.05. *p ≤ 0.05, **p ≤ 0.01, ***p ≤ 0.001, ****p ≤ 0.0001. OD, optical density.

To test whether changes in glucose homeostasis in 8‐week‐old TKO mice are driven by defects in insulin signaling activation, we measured changes in insulin signaling in various tissues by measuring AKT phosphorylation status after insulin injection. Although fasting glucose levels were significantly increased in TKO mice after a 16‐h fast, blood glucose levels of both TKO and WT decreased to similar levels 20 min after insulin injection (Figure 4A), suggesting TKO mice are able to properly respond to insulin. Consistent with these findings, we did not observe any changes in AKT phosphorylation status between WT and TKO mice 20 min after insulin injection within gastrocnemius muscle (Figure 4B,C), iWAT (Figure 4B,D), BAT (Figure 4B,E), or liver (Figure 4B,F). Our data indicate that the impairments in glucose homeostasis observed in TKO mice are likely independent of changes in insulin signaling or systemic changes in insulin sensitivity, as also indicated by normal ITT (Figure 2Q,R).

### 3.5 Simultaneous Deletion of GDF15 and FGF21 in OPA1 BKO Mice Significantly Blunted Resistance to DIO

**FIGURE 5:**
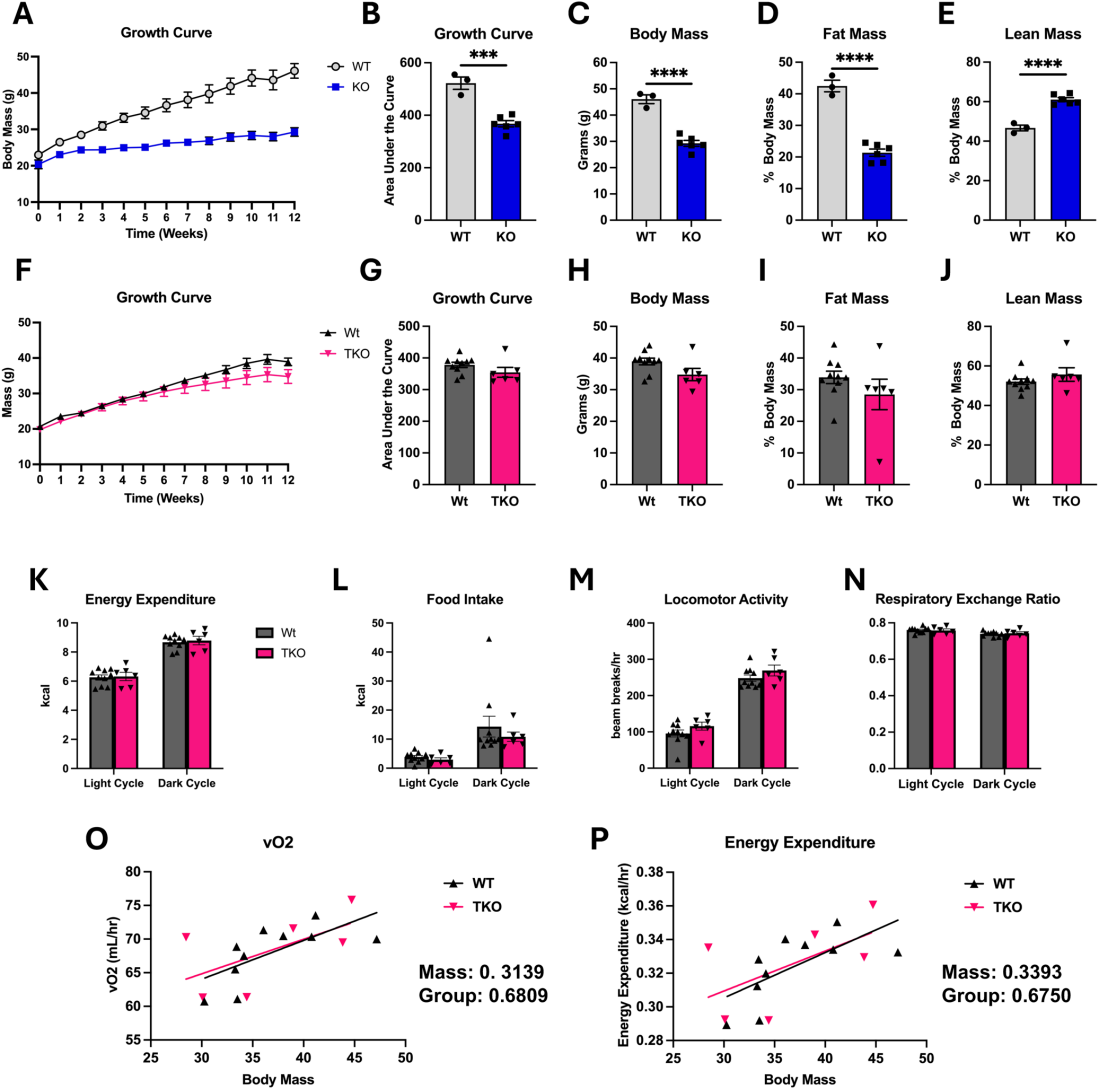
Simultaneous deletion of GDF15 and FGF21 in OPA1 BKO mice completely prevented resistance to diet‐induced obesity. In panels A–E, data collected in OPA1 BKO male mice and WT controls fed HFD for 12 weeks (*n* = 3 WT; *n* = 6 OPA1 BKO). (A) Body mass growth curve. (B) AUC for growth curve. (C–E) Body composition data (*n* = 3 WT; *n* = 6 OPA1 BKO). (C) Body mass. (D) Percent fat mass to body weight. (E) Percent lean mass to body weight. In panels F–Q, data collected in TKO male mice and WT controls fed HFD for 12 weeks (*n* = 10 WT; *n* = 6 TKO). (F) Body mass growth curve. (G) AUC for growth curve. (H–J) Body composition data. (H) Body mass. (I) Percent fat mass to body weight. (J) Percent lean mass to body weight. Indirect calorimetry data represented as the average for the light and dark cycles during the last 48 h of data recording. (K) Energy expenditure. (L) Food intake. (M) Locomotor activity. (N) Respiratory exchange ratio. (O) Regression plot of oxygen consumption as a function of body mass. (P) Regression plot of energy expenditure as a function of body mass. Data expressed as mean ± SEM. Significant differences determined by Student's *t*‐test, two‐way ANOVA followed by Tukey's multiple‐comparison test, or ANCOVA for the group effect and by using a significance level of *p* ≤ 0.05. ***p ≤ 0.001, ****p ≤ 0.0001.

Our previous studies showed that OPA1 BKO mice are completely resistant to DIO, a phenotype that was lost in mice lacking both OPA1 and ATF4 in BAT (OPA1/ATF4 DKO) [9]. Because OPA1/ATF4 DKO mice also lacked the induction of FGF21 and GDF15 [9, 10], we hypothesized that FGF21 and GDF15 may exert synergistic effects contributing to leanness in OPA1 BKO mice fed HFD. To test this hypothesis, we fed TKO and their WT littermate controls HFD (60% calories from fat) for 12 weeks. For comparison purposes, we first recapitulated our data in a new cohort of OPA1 BKO mice. As previously reported, this new cohort of OPA1 BKO mice had reduced body mass (Figure 5A–C), fat mass percentage (Figure 5D), and total fat mass (Figure S3A), as well as increased lean mass percentage (Figure 5E) and decreased total lean mass (Figure S3B), after 12 weeks on HFD [9]. Conversely, TKO mice had no changes in body mass over 12 weeks on HFD (Figure 5F–H, Figure S3C,D). Both total and percent fat mass (Figure 5I, Figure S3C) and lean mass (Figure 5J, Figure S3D) were unchanged between TKO mice and their WT controls. Importantly, final body weight after HFD was significantly increased in TKO mice when compared to OPA1 BKO mice (Figure S3E). We previously showed that OPA1 BKO mice had increased energy expenditure and higher food intake, locomotor activity, and respiratory exchange ratio relative to WT mice while on HFD [9, 10]. However, in TKO mice, we observed no changes in metabolic parameters such as energy expenditure (Figure 5K), food intake (Figure 5L), locomotor activity (Figure 5M), or respiratory exchange ratio (Figure 5N). Importantly, ANCOVA of oxygen consumption rates (Figure 5O) and energy expenditure (Figure 5P) as a function of body mass showed no significant group effect, confirming that combined deletion of FGF21 and GDF15 in OPA1 BKO mice completely blunts the increase in resting metabolic rates that drives the lean phenotype in these mice.

### 3.6 TKO Mice Lack the Protection Against Diet‐Induced Glucose Intolerance and Insulin Resistance Observed in OPA1 BKO Mice

**FIGURE 6:**
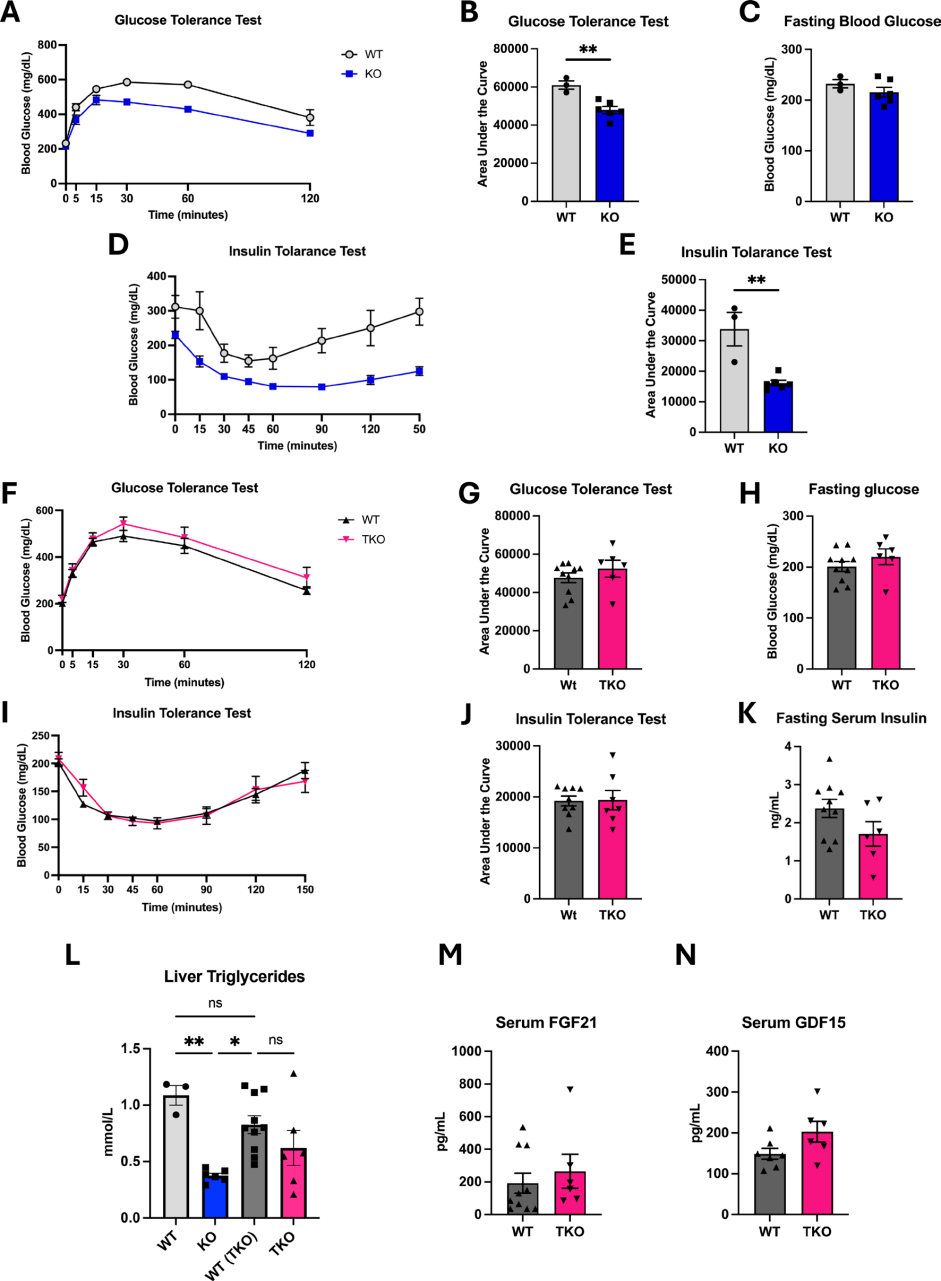
TKO mice lack the protection against diet‐induced glucose intolerance and insulin resistance observed in OPA1 BKO mice. In panels A–E and L, data collected in OPA1 BKO male mice and their WT controls fed HFD for 12 weeks (*n* = 3 WT; *n* = 6 OPA1 BKO). (A) Glucose tolerance test. (B) AUC for glucose tolerance test. (C) Fasting blood glucose levels. (D) Insulin tolerance test. (E) AUC for insulin tolerance test. In panels F–N, data collected in TKO male mice and WT controls fed HFD for 12 weeks (*n* = 10 WT; *n* = 6 TKO). (F) Glucose tolerance test. (G) AUC for glucose tolerance test. (H) Fasting blood glucose levels. (I) Insulin tolerance test. (J) AUC for insulin tolerance test. (K) Fasting serum insulin levels. (L) Liver triglycerides. (M) FGF21 serum levels. (N) GDF15 serum levels (*n* = 7 WT; *n* = 6 TKO). Data expressed as mean ± SEM. Significant differences determined by Student's *t*‐test using a significance level of *p* ≤ 0.05. **p ≤ 0.01.

To assess glucose homeostasis and insulin resistance in mice fed HFD, we first used a new cohort of OPA1 BKO mice to recapitulate our previous findings [9]. As in our previous work, we observed improved glucose tolerance (Figure 6A,B), with no changes in fasting blood glucose levels (Figure 6C) after 12 weeks of HFD. Insulin sensitivity was also markedly improved in OPA1 BKO mice after 12 weeks on HFD, as shown by the decreased AUC for ITT (Figure 6D,E). Conversely, TKO mice lacked any improvement in glucose homeostasis (Figure 6F–H) or insulin sensitivity (Figure 6I,J) after 12 weeks on HFD. Fasting serum insulin levels (Figure 6K) were also unchanged between WT and TKO male mice. Notably, hepatic triglyceride accumulation was significantly attenuated in OPA1 BKO mice, which was prevented in TKO mice (Figure 6L). Finally, serum FGF21 (Figure 6M) and GDF15 (Figure 6N) levels after 12 weeks of HFD were similar between WT and TKO mice.

Our data were confirmed in TKO female mice; body weight (Figure S3F–H), body composition (Figure S3I,J), glucose homeostasis (Figure S3K–M), and insulin sensitivity (Figure S3N,O) were similar between WT and TKO female mice after 12 weeks on HFD. Fasting serum insulin (Figure S3P) and liver triglyceride levels (Figure S3Q) were also unchanged between genotypes in female mice. Together, our data show that FGF21 and GDF15 act together to regulate changes in glucose homeostasis and insulin sensitivity in OPA1 BKO mice under obesogenic conditions.

## 4 Discussion

Obesity is a chronic and multifactorial disease that remains a key modifiable risk factor for several comorbidities, including cardiovascular disease and diabetes mellitus [1]. The discovery of functional BAT in adult humans has opened new opportunities to investigate its potential therapeutic applications for the treatment of obesity and associated metabolic disorders. Importantly, the role of BAT extends beyond thermogenesis and includes the regulation of glucose and lipid homeostasis via the release of mediators that communicate with other cells and tissues [2]. Our earlier studies revealed that the secretion of FGF21 and GDF15 from BAT plays an important role in improving metabolism in OPA1 BKO mice. We found that FGF21 drove compensatory browning of white adipose tissue and promoted leanness in OPA1 BKO mice fed a regular chow diet [9], while GDF15, but not FGF21, contributed to DIO resistance in OPA1 BKO mice [10]. In the present study, we tested the hypothesis that, in addition to their independent effects, FGF21 and GDF15 play synergistic roles in promoting the systemic metabolic improvements observed in OPA1 BKO mice.

FGF21 is a member of the fibroblast growth factor family, which can be released into the bloodstream to act as a hormone. FGF21 is primarily produced by the liver, but it can also be induced in various other tissues, including adipose tissue, in response to nutritional and cellular stress. FGF21 actions have been shown to enhance insulin sensitivity and increase energy expenditure in preclinical models via peripheral and central mechanisms, respectively [22]. Similarly, GDF15 is a small peptide member of the transforming growth factor‐β superfamily that can be secreted into circulation to exert systemic effects. It can be induced in various tissues, including liver, BAT, and skeletal muscle in response to stress and disease conditions such as mitochondrial disease and obesity [23]. GDF15 functions by binding to its central receptor, GFRAL, to repress food intake and increase energy expenditure, thereby promoting DIO resistance in preclinical models [24]. A recent study demonstrated that the combined whole‐body deletion of FGF21 and GDF15 does not exacerbate DIO, but results in significant worsening of hepatic steatosis and insulin resistance relative to that observed in GDF15 single knockout mice, indicating that these endocrine factors might act synergistically to protect against obesity‐induced metabolic derangements [25].

Here we show that in the OPA1 BKO background, combined deletion of *Fgf21* and *Gdf15* in BAT led to impaired glucose homeostasis despite no changes in body weight or body composition in young chow‐fed mice. Interestingly, neither *Gdf15* nor *Fgf21* deletion alone affected baseline glucose homeostasis in OPA1 BKO mice [9, 10], whereas OPA1/ATF4 DKO mice, which lacked the induction in FGF21 and GDF15, experienced similar defects in glucose tolerance as TKO mice. These results suggest that maintenance of normal glucose metabolism in OPA1 BKO mice requires the combined effects of FGF21 and GDF15. We also show that OPA1 BKO mice have improved insulin sensitivity, which was absent in TKO mice. Since we did not measure insulin sensitivity in OPA1/FGF21 and OPA1/GDF15 DKO mice under baseline conditions, we are unable to ascertain whether this is the result of independent effects of GDF15 or FGF21 or whether synergistic actions are required. Interestingly, insulin signaling was preserved in TKO mice, suggesting that the impairments in glucose metabolism observed in these mice might have occurred due to defects in insulin secretion. Indeed, recent studies suggest that GDF15 might prevent β‐cell death and reduce inflammation [26, 27, 28]. Moreover, FGF21 has been shown to be protective against lipotoxicity‐induced apoptosis and β‐cell dysfunction [29, 30]. Future studies investigating β‐cell function in TKO mice should shed light on the protective roles of FGF21 and GDF15 and could provide an explanation for the mild impairments in glucose homeostasis observed in these mice.

Importantly, simultaneous deletion of *Fgf21* and *Gdf15* in BAT significantly attenuated the resistance to DIO and insulin resistance in OPA1 BKO mice. These results contrast with studies in OPA1/FGF21 DKO mice, which retained the lean phenotype [9] and surpassed the effects of *Gdf15* deletion alone, which only partially prevented DIO resistance [10]. Of note, our results in TKO mice recapitulate our data in OPA1/ATF4 DKO mice [9], reinforcing the idea that the combined actions of FGF21 and GDF15 are required to promote leanness in OPA1 BKO mice. One limitation in our study design is the lack of direct comparisons between OPA1 BKO and TKO mice. This is because we opted to compare our OPA1 BKO and TKO mutants to their respective age‐matched littermate controls. Unfortunately, the response of the different WT groups to DIO was slightly different, with the WT for the TKO being slightly more resistant to weight gain than the WT for the OPA1 BKO colony. Nonetheless, body weight was still significantly different between OPA1 BKO and TKO mice after HFD, reinforcing the need for FGF21 and GDF15 to drive this phenotype. Our data suggest that FGF21 and GDF15 may potentiate each other's effects on energy expenditure during DIO, via undetermined molecular mechanisms.

## 5 Conclusion

We demonstrate that FGF21 and GDF15 act together to preserve glucose homeostasis in unstressed OPA1 BKO mice. Moreover, FGF21 and GDF15 synergistic effects are required to mediate the resistance to DIO when OPA1 is deleted in BAT, likely by increasing energy expenditure. Future preclinical studies using both FGF21 and GDF15 analogues in combination should shed light on their therapeutic potential to attenuate obesity and associated comorbidities. Furthermore, determining whether these factors also play any synergistic roles in BAT physiology would advance our understanding of FGF21 and GDF15 biology.

## Conflicts of Interest

The authors declare no conflicts of interest.

## Supporting information


**Figure S1:** (A) Data collected from 8‐week‐old OPA1 BKO Males. mRNA expression of thermogenic genes normalized to *Tbp* expression (*n* = 6 WT; *n* = 6 OPA1 BKO). (B–G) Data collected from 8‐week‐old OPA1 BKO females raised at thermoneutral conditions. mRNA expression of thermogenic genes normalized to *Gapdh* expression (*n* = 3–5 WT; *n* = 3–5 OPA1 BKO). (C) Relative mRNA expression of *Fgf21* in BAT (*n* = 6 WT; *n* = 4 OPA1 BKO). (D) Relative mRNA expression of *Gdf15* in BAT (*n* = 6 WT; *n* = 4 OPA1 BKO). (E) Serum levels of FGF21 in ad libitum‐fed mice (*n* = 6 WT; *n* = 6 OPA1 BKO). (F) Serum levels of GDF15 in ad libitum‐fed mice (*n* = 10 WT; *n* = 6 OPA1 BKO). (G) mRNA expression of thermogenic genes in iWAT normalized to *Gapdh* expression (*n* = 4 WT; *n* = 3 OPA1 BKO). Data are expressed as means ± SEM. Significant differences determined by Student’s *t*‐test using a significance level of *p* ≤ 0.05. *p ≤ 0.05.

**Figure S2:** (A, B) Data collected in 8‐week‐old male OPA1 BKO and WT control mice fed chow diet (*n* = 10 WT; *n* = 12 OPA1 BKO). (A) Fat mass. (B) Lean mass. (C–E) Data collected in 7‐week‐old female OPA1 BKO and WT control mice fed chow diet. (C) Body mass (*n* = 4 WT; *n* = 6 OPA1 BKO). (D) Fat mass (*n* = 3 WT; *n* = 5 OPA1 BKO). (E) Lean mass (*n* = 4 WT; *n* = 6 OPA1 BKO). (F–K) Data collected in 8‐week‐old male WT and TKO mice fed chow diet. (F) Fat mass (*n* = 40 WT; *n* = 41 TKO). (G) Lean mass (*n* = 40 WT; *n* = 41 TKO). (H) Liver mass (*n* = 17 WT; *n* = 13 TKO). (I) BAT mass (*n* = 17 WT; *n* = 13 TKO). (J) iWAT mass (*n* = 17 WT; *n* = 13 TKO). (K) gWAT mass (*n* = 17 WT; *n* = 13 TKO). (L–N) Data collected in 20‐week‐old OPA1/FGF21 DKO male mice fed chow diet. (L) Glucose tolerance test (*n* = 13 WT; *n* = 8 OPA1/FGF21). (M) AUC for glucose tolerance test (*n* = 13 WT; *n* = 8 OPA1/FGF21). (N) Fasting blood glucose levels (*n* = 13 WT; *n* = 8 OPA1/FGF21). (O–Q) Data collected in 8‐week‐old OPA1/ATF4 DKO male mice fed chow diet. (O) Glucose tolerance test (*n* = 5 WT; *n* = 5 OPA1/ATF4). (P) AUC for glucose tolerance test (*n* = 5 WT; *n* = 5 OPA1/ATF4). (Q) 4‐h fasting blood glucose levels (*n* = 5 WT; *n* = 5 OPA1/ATF4). (R–W) Data collected in 8‐week‐old TKO female mice fed chow diet. (R) Body mass (*n* = 15–19/gp). (S) Percent fat mass to body weight (*n* = 19 WT; *n* = 15 TKO). (T) Percent lean mass to body weight (*n* = 19 WT; *n* = 15 TKO). (U) Glucose tolerance test (*n* = 7 WT; *n* = 9 TKO). (V) AUC for glucose tolerance test (*n* = 7 WT; *n* = 9 TKO). (W) Fasting blood glucose levels (*n* = 7 WT; *n* = 9 TKO). Data expressed as mean ± SEM. Significant differences determined by Student’s *t*‐test using a significance level of *p* ≤ 0.05. *p ≤ 0.05, **p ≤ 0.01.

**Figure S3:** (A, B, E) Data collected in OPA1 BKO male mice fed HFD for 12 weeks (*n* = 3 WT; *n* = 6 OPA1 BKO). (A) Fat mass. (B) Lean mass. (C–E) TKO male mice fed HFD for 12 weeks (*n* = 10 WT; *n* = 6 TKO). (C) Fat mass. (D) Lean mass. (C) Body mass after 12 weeks of HFD (*n* = 6 OPA1 BKO; *n* = 6 TKO). (F–Q) Data collected in TKO female mice fed HFD for 12 weeks (*n* = 5 WT; *n* = 8 TKO). (F) Body mass growth curve. (G) AUC for growth curve. (H–J) Body composition. (H) Body mass. (I) Percent fat mass to body weight. (J) Percent lean mass to body weight. (K) Glucose tolerance test. (L) AUC for glucose tolerance test. (M) Fasting blood glucose levels. (N) Insulin tolerance test. (O) AUC for insulin tolerance test. (P) Fasting serum insulin levels. (Q) Liver triglycerides. Data expressed as mean ± SEM. Significant differences determined by Student’s *t*‐test using a significance level of *p* ≤ 0.05. *p ≤ 0.05, ****p ≤ 0.0001.

## Data Availability

The data that support the findings of this study are available from the corresponding author upon reasonable request.
