# Supplementary figures and images for: FGF21 and GDF15 Act Synergistically to Regulate Systemic Metabolic Homeostasis in Mice Lacking OPA1 in Thermogenic Adipocytes
Source: Obesity (Silver Spring). 2025 Sep 2;33(10):1909–20. doi: 10.1002/oby.70004 (PMC12477105; doi:10.1002/oby.70004)

**A**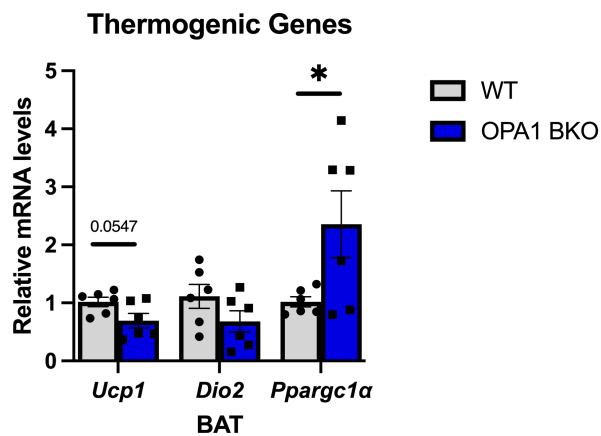**B**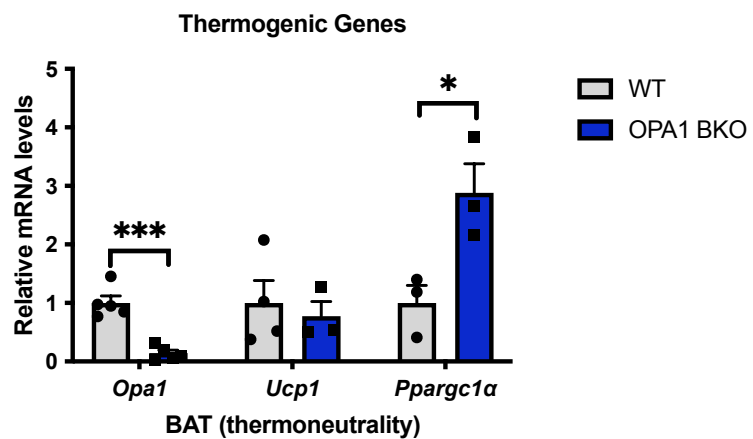**C**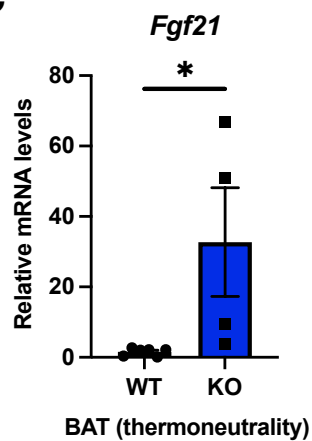**D**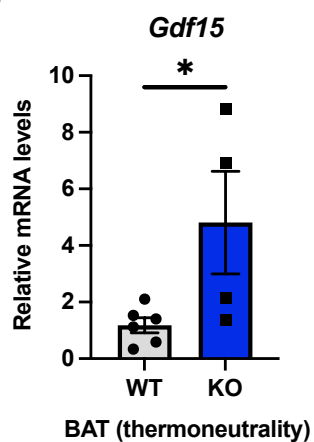**E**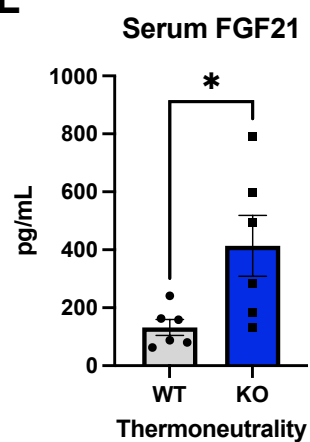**F**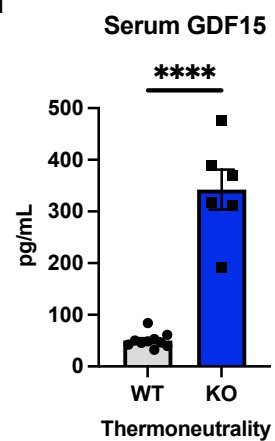**G**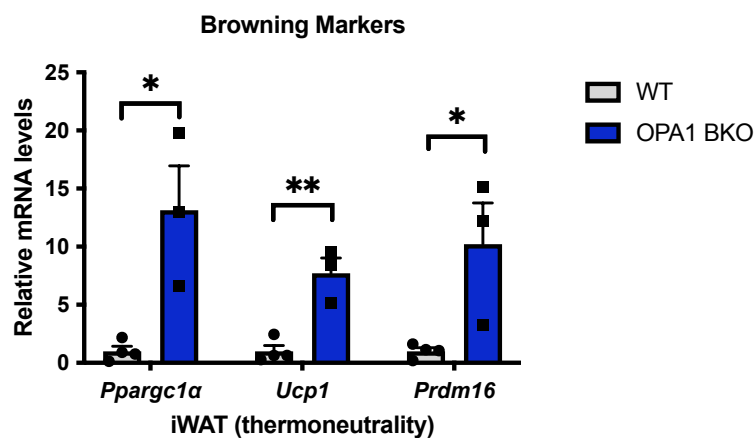

Supplement: Supplementary file 1 — Figure S1: (A) Data collected from 8‐week‐old OPA1 BKO Males. mRNA expression of thermogenic genes normalized to Tbp expression (n = 6 WT; n = 6 OPA1 BKO). (B–G) Data collected from 8‐week‐old OPA1 BKO females raised at thermoneutral conditions. mRNA expression of thermogenic genes normalized to Gapdh expression (n = 3–5 WT; n = 3–5 OPA1 BKO). (C) Relative mRNA expression of Fgf21 in BAT (n = 6 WT; n = 4 OPA1 BKO). (D) Relative mRNA expression of Gdf15 in BAT (n = 6 WT; n = 4 OPA1 BKO). (E) Serum levels of FGF21 in ad libitum‐fed mice (n = 6 WT; n = 6 OPA1 BKO). (F) Serum levels of GDF15 in ad libitum‐fed mice (n = 10 WT; n = 6 OPA1 BKO). (G) mRNA expression of thermogenic genes in iWAT normalized to Gapdh expression (n = 4 WT; n = 3 OPA1 BKO). Data are expressed as means ± SEM. Significant differences determined by Student’s t‐test using a significance level of p ≤ 0.05. *p ≤ 0.05. [file OBY-33-1909-s002.pdf]

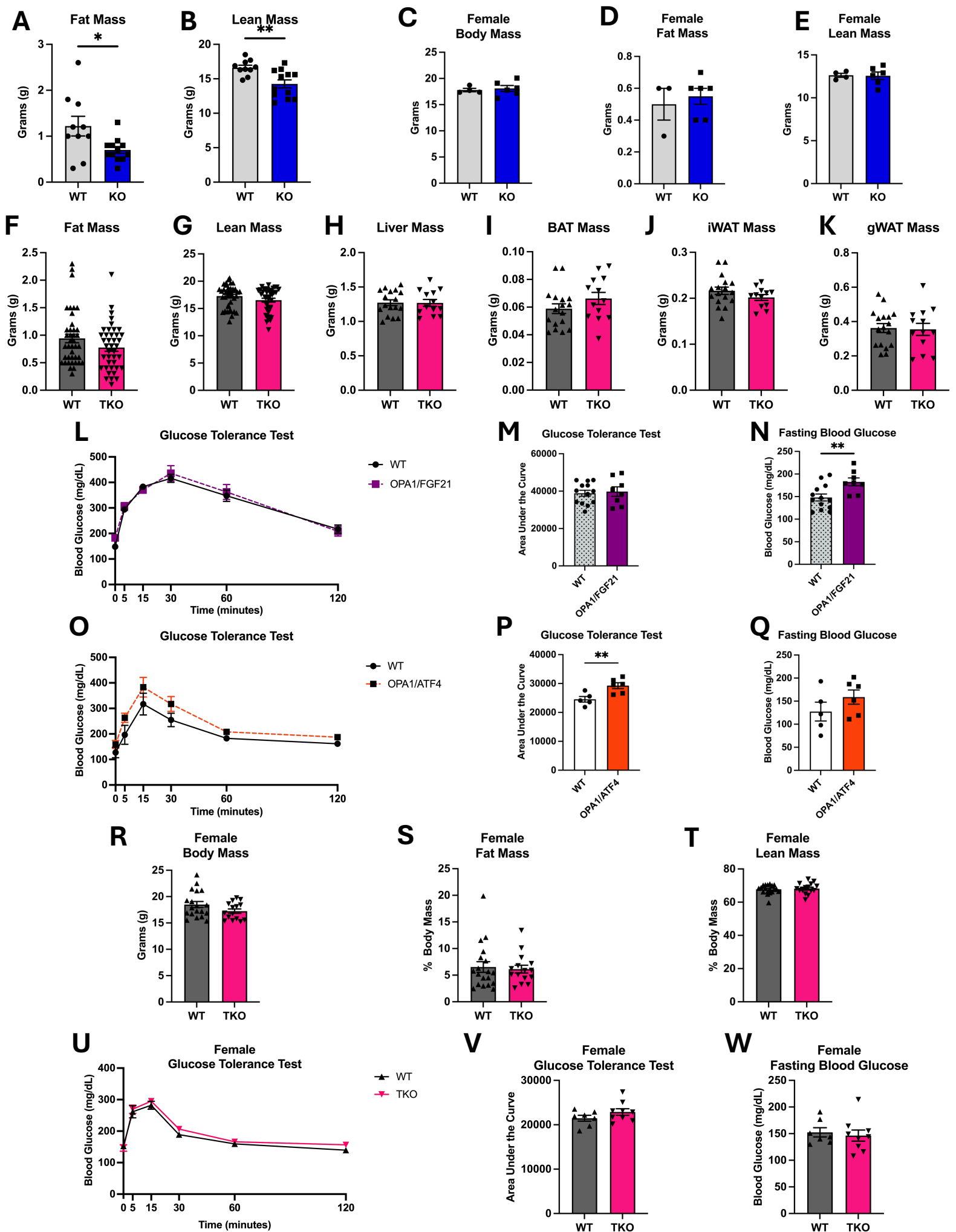

Supplemental Figure 2

Supplement: Supplementary file 2 — Figure S2: (A, B) Data collected in 8‐week‐old male OPA1 BKO and WT control mice fed chow diet (n = 10 WT; n = 12 OPA1 BKO). (A) Fat mass. (B) Lean mass. (C–E) Data collected in 7‐week‐old female OPA1 BKO and WT control mice fed chow diet. (C) Body mass (n = 4 WT; n = 6 OPA1 BKO). (D) Fat mass (n = 3 WT; n = 5 OPA1 BKO). (E) Lean mass (n = 4 WT; n = 6 OPA1 BKO). (F–K) Data collected in 8‐week‐old male WT and TKO mice fed chow diet. (F) Fat mass (n = 40 WT; n = 41 TKO). (G) Lean mass (n = 40 WT; n = 41 TKO). (H) Liver mass (n = 17 WT; n = 13 TKO). (I) BAT mass (n = 17 WT; n = 13 TKO). (J) iWAT mass (n = 17 WT; n = 13 TKO). (K) gWAT mass (n = 17 WT; n = 13 TKO). (L–N) Data collected in 20‐week‐old OPA1/FGF21 DKO male mice fed chow diet. (L) Glucose tolerance test (n = 13 WT; n = 8 OPA1/FGF21). (M) AUC for glucose tolerance test (n = 13 WT; n = 8 OPA1/FGF21). (N) Fasting blood glucose levels (n = 13 WT; n = 8 OPA1/FGF21). (O–Q) Data collected in 8‐week‐old OPA1/ATF4 DKO male mice fed chow diet. (O) Glucose tolerance test (n = 5 WT; n = 5 OPA1/ATF4). (P) AUC for glucose tolerance test (n = 5 WT; n = 5 OPA1/ATF4). (Q) 4‐h fasting blood glucose levels (n = 5 WT; n = 5 OPA1/ATF4). (R–W) Data collected in 8‐week‐old TKO female mice fed chow diet. (R) Body mass (n = 15–19/gp). (S) Percent fat mass to body weight (n = 19 WT; n = 15 TKO). (T) Percent lean mass to body weight (n = 19 WT; n = 15 TKO). (U) Glucose tolerance test (n = 7 WT; n = 9 TKO). (V) AUC for glucose tolerance test (n = 7 WT; n = 9 TKO). (W) Fasting blood glucose levels (n = 7 WT; n = 9 TKO). Data expressed as mean ± SEM. Significant differences determined by Student’s t‐test using a significance level of p ≤ 0.05. *p ≤ 0.05, **p ≤ 0.01. [file OBY-33-1909-s001.pdf]

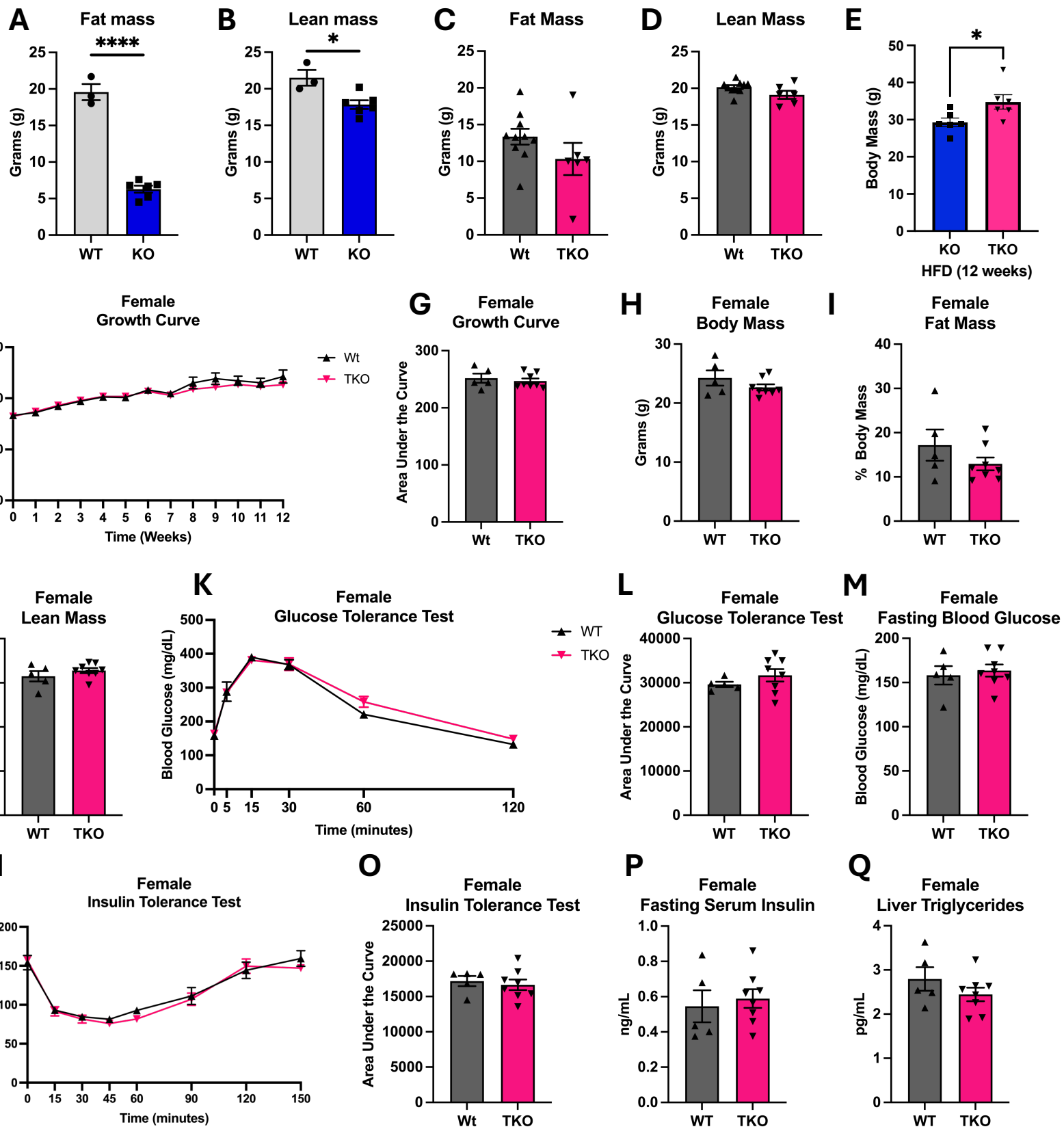

Supplemental Figure 3

Supplement: Supplementary file 3 — Figure S3: (A, B, E) Data collected in OPA1 BKO male mice fed HFD for 12 weeks (n = 3 WT; n = 6 OPA1 BKO). (A) Fat mass. (B) Lean mass. (C–E) TKO male mice fed HFD for 12 weeks (n = 10 WT; n = 6 TKO). (C) Fat mass. (D) Lean mass. (C) Body mass after 12 weeks of HFD (n = 6 OPA1 BKO; n = 6 TKO). (F–Q) Data collected in TKO female mice fed HFD for 12 weeks (n = 5 WT; n = 8 TKO). (F) Body mass growth curve. (G) AUC for growth curve. (H–J) Body composition. (H) Body mass. (I) Percent fat mass to body weight. (J) Percent lean mass to body weight. (K) Glucose tolerance test. (L) AUC for glucose tolerance test. (M) Fasting blood glucose levels. (N) Insulin tolerance test. (O) AUC for insulin tolerance test. (P) Fasting serum insulin levels. (Q) Liver triglycerides. Data expressed as mean ± SEM. Significant differences determined by Student’s t‐test using a significance level of p ≤ 0.05. *p ≤ 0.05, ****p ≤ 0.0001. [file OBY-33-1909-s003.pdf]
